# Metazen – metadata capture for metagenomes

**DOI:** 10.1186/1944-3277-9-18

**Published:** 2014-12-08

**Authors:** Jared Bischof, Travis Harrison, Tobias Paczian, Elizabeth Glass, Andreas Wilke, Folker Meyer

**Affiliations:** 1 Computation Institute, University of Chicago, 5735 S Ellis Ave, Chicago, IL 60637, USA; 2 Mathematics and Computer Science Division, Argonne National Laboratory, 9700 S. Cass Ave, Argonne, IL 60439, USA; 3 Biological Sciences Division, Argonne National Laboratory, 9700 S. Cass Ave, Argonne, IL 60439, USA

**Keywords:** Metadata, Metagenomics, Collection, Software

## Abstract

**Background:**

As the impact and prevalence of large-scale metagenomic surveys grow, so does the acute need for more complete and standards compliant metadata. Metadata (data describing data) provides an essential complement to experimental data, helping to answer questions about its source, mode of collection, and reliability. Metadata collection and interpretation have become vital to the genomics and metagenomics communities, but considerable challenges remain, including exchange, curation, and distribution.

Currently, tools are available for capturing basic field metadata during sampling, and for storing, updating and viewing it. Unfortunately, these tools are not specifically designed for metagenomic surveys; in particular, they lack the appropriate metadata collection templates, a centralized storage repository, and a unique ID linking system that can be used to easily port complete and compatible metagenomic metadata into widely used assembly and sequence analysis tools.

**Results:**

Metazen was developed as a comprehensive framework designed to enable metadata capture for metagenomic sequencing projects. Specifically, Metazen provides a rapid, easy-to-use portal to encourage early deposition of project and sample metadata.

**Conclusions:**

Metazen is an interactive tool that aids users in recording their metadata in a complete and valid format. A defined set of mandatory fields captures vital information, while the option to add fields provides flexibility.

## Background

As the impact and prevalence of large-scale metagenomic surveys grow, so does the acute need for more complete and standards compliant metadata. Metadata (data describing data) provides an essential complement to experimental data, helping to answer questions about its source, mode of collection, and reliability. Metadata collection and interpretation have become vital to the genomics and metagenomics communities, but considerable challenges remain, including exchange, curation, and distribution. Currently, tools are available for capturing basic field metadata during sampling and for storing, updating, and viewing it. Unfortunately, these tools are not specifically designed for metagenomic surveys, as they lack the appropriate metadata collection templates, a centralized storage repository, and a unique ID linking system that can be used to easily port complete and compatible metagenomic metadata into widely used assembly and sequence analysis tools. Metadata are frequently incomplete or are recorded by using widely varying ontologies, dramatically decreasing the value of data and limiting the power of analyses. Further, correcting compatibility issues is a time-consuming, manually performed task. Although much of the underpinning infrastructure and software already exist that could remedy this problem, the tools and technologies have not yet been brought together in a way that makes the entry, merging, and transfer of such data simple.

The Genomic Standards Consortium (GSC, [1]) has developed widely accepted metadata standards for genomic, metagenomic, and amplicon (e.g., 16S rRNA) sequence datasets [2-4]. These standards have been, and continue to be, developed within the GCDML framework [3], which is both modular and extensible. The framework consists of checklists for any type of genomic data as well as additional environmental packages. For example, the checklist for metagenomic data, combined with the package of metadata describing the observations of a particular environment, is a powerful means of comprehensively and consistently reporting all metadata of a particular metagenomic sample and experiment.

Capturing metadata early and in an electronic format is widely viewed as the solution to the current metadata crisis. While many software systems provide metadata capturing support (MG-RAST [5], GOLD [6], VAMPS [7], IMG/M [8], CAMERA [9], QIIME [10], ISA tools [11], and RightFielder [12]), adding metadata at the time of sequence upload to an analysis resource is viewed by nearly all users as an additional hurdle that they need to overcome as quickly as possible. The fact that less than 10% of all data sets in the MG-RAST repository had complete minimal metadata according to GSC standards highlights the nature of the crisis.

Public sequence repositories support upload of metadata as structured comments (e.g. NCBI’s GenBank), however, they do not provide any tooling to the users. They instead rely on upstream tools to ensure metadata completeness and correctness.

Metazen (Meta for metadata and Zen for the Japanese word for “complete”) is a comprehensive framework designed to enable metadata capture for metagenomic sequencing projects [13]. Specifically, Metazen provides a rapid, easy-to-use portal to encourage early deposition of project and sample metadata. It is a stand-alone tool that provides the ability to validate spreadsheets against a number of controlled vocabularies and other “gatekeepers” (e.g. regular expressions to ensure controlled syntax) to ensure document compliance.

## Results and discussion

**Figure 1:**
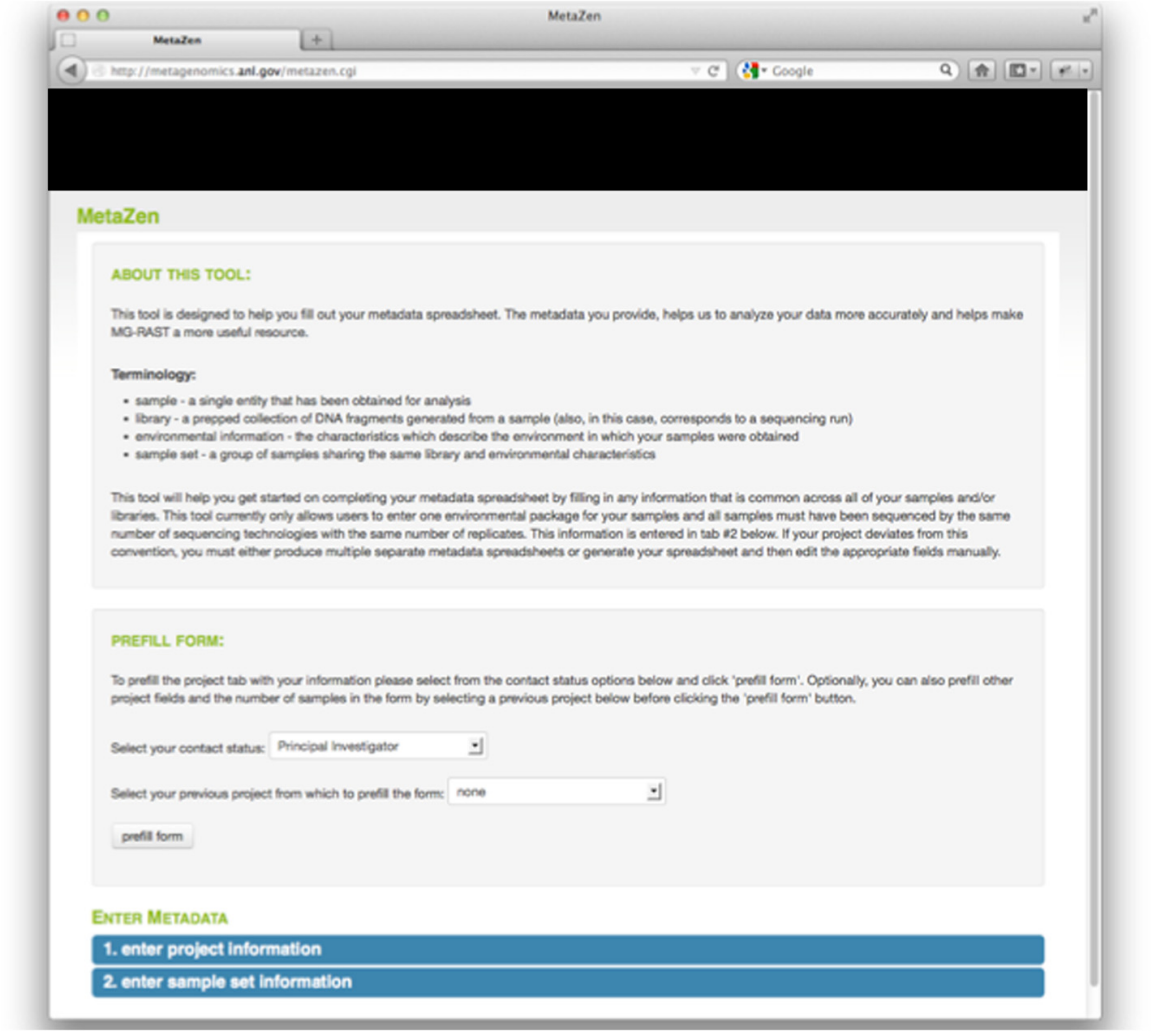
Metazen login page.

Metazen is an interactive tool that aids users in recording their metadata in a complete and valid format. A defined set of mandatory fields captures vital information, while the option to add fields provides flexibility. Project and user-level information is stored so users can reload that information without having to enter it repeatedly. Entry into Metazen starts with the login page (Figure 1).

**Figure 2:**
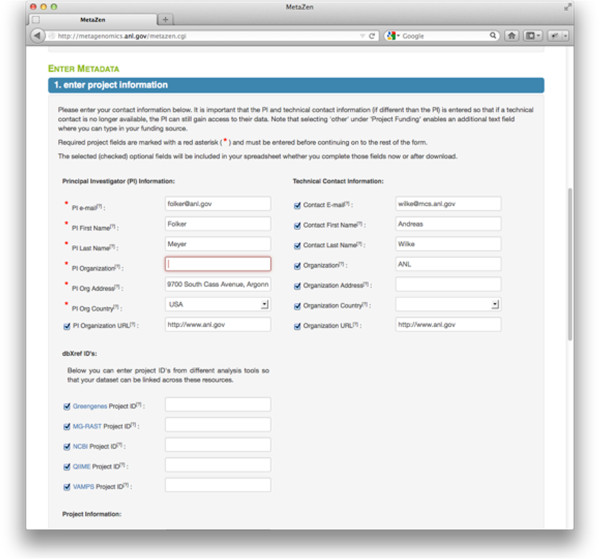
**Metadata form.** Stored information can be used to prefill fields. Not all fields are required, but they are validated. Note that in this example the PI Organization is a required field that was left blank.

**Figure 3:**
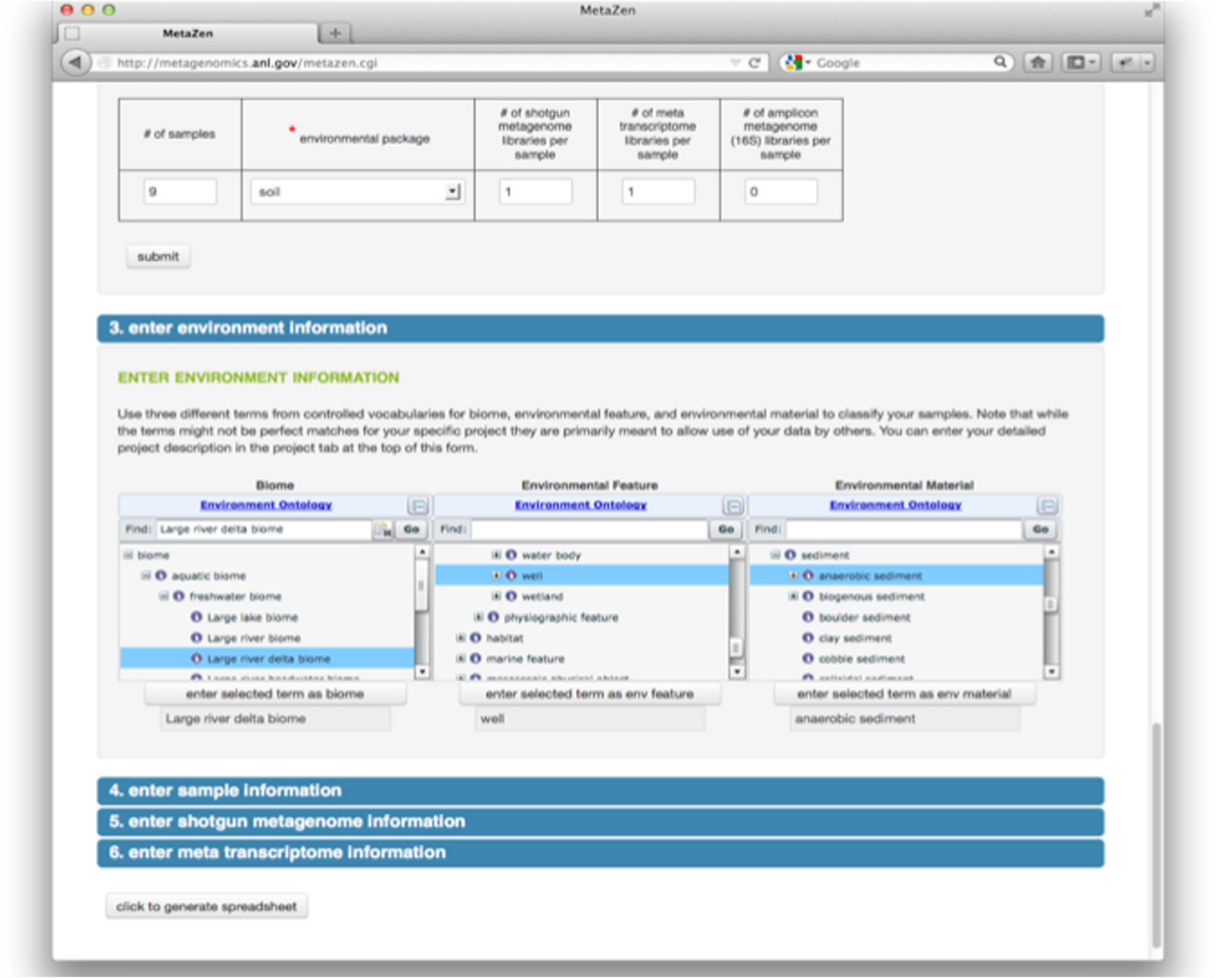
Viewing controlled vocabularies in Metazen.

Metazen presents a simple, user-friendly web interface that has a limited number of mandatory terms (following the GSC standards). The web site strives to make the tedious task of adding structured data as user-friendly as possible. This is accomplished by providing drop down-menus, providing documentation, comments and examples and finally using spreadsheet technology the users are familiar with.Project-level information can be prefilled (if desired by the user) from existing projects in the system. Figure 2 shows the fields that have been prefilled by the stored user information, required fields versus optional fields, and a highlighted field that indicates how the user is informed of missing required fields or invalid data entry.Figure 3 shows where users can pick an environmental package (top region of screenshot). Also, complex, controlled vocabularies are more easily navigated and chosen through Bioportal widgets. Selection of controlled vocabulary terms that help explain data to third parties is facilitated via widgets imported from Bioontology.org. Users can explore the existing controlled vocabulary terms and prefill the spreadsheet.

### Impact on metadata collection since using Metazen

While prior to the introduction of Metazen, only 10% of all data sets in MG-RAST had fully GSC compliant metadata. After the introduction of Metazen the number has become significantly higher. We now have well over 40,000 data sets that have compliant metadata.

### Editing and validating metadata

Once users are finished entering their metadata into Metazen, a spreadsheet can be generated for download and further editing. Often, users will want to edit their spreadsheet manually if they have many samples and/or sequencing runs for which they would like to enter specific metadata on a per sample or per run basis. Having both the web and spreadsheet to interact with provides users with guidance to follow standards and the flexibility to extend and modify their metadata. Once they are finished editing their metadata, a validation tool helps users both identify metadata errors and ensure that the submitted spreadsheet contains the vital (required) metadata fields and completely valid data (see Methods for more details).

We have created architecture, called Metazen Collect, for a new, user-friendly metadata-capture software application. Metazen Collect will be accessible via the Internet and with commonly used smart phone operating systems (Android and Apple IOs). It has the capability to easily export metadata files in Excel.

## Conclusions

Metazen provides researchers with a tool to collect and contribute metadata easily, using community data standards and controlled vocabularies. Highlights and unique attributes of this software include the following:

• Project-level information can be saved for reuse when creating the metadata for a new project.

• Required fields help capture, at a minimum, the most vital information.

• Field validation at this interactive site helps ensure the integrity of the metadata and guides the user through what is often a difficult endeavor.

• A strict format requirement for various data types provides the ability to search metadata fields at a later time.

• Controlled vocabularies are easily navigated and chosen through dropdown menus and BioPortal bioontology [14] widgets.

• Searching the Google maps API can help users obtain the geographic coordinates of where their samples were obtained.

• In order to assist third parties, the metadata created is available for download in MS Excel format from the MG-RAST download pages and via the web services API (Metazen uses the MG-RAST API to retrieve project-level metadata and our metadata template).

The Metazen architecture allows for easy extensions into new metadata packages and new data types (like GWAS, metaproteomes, or microarray data). Metazen uses GSC metadata standards as “data” and not as a fixed part of the software framework. This allows for updated or new metadata packages to be “plugged-in” easily. This also allows users to create new subsets of terms (and the corresponding controlled vocabulary) to capture more data on their field of study.

## Methods

**Figure 4:**
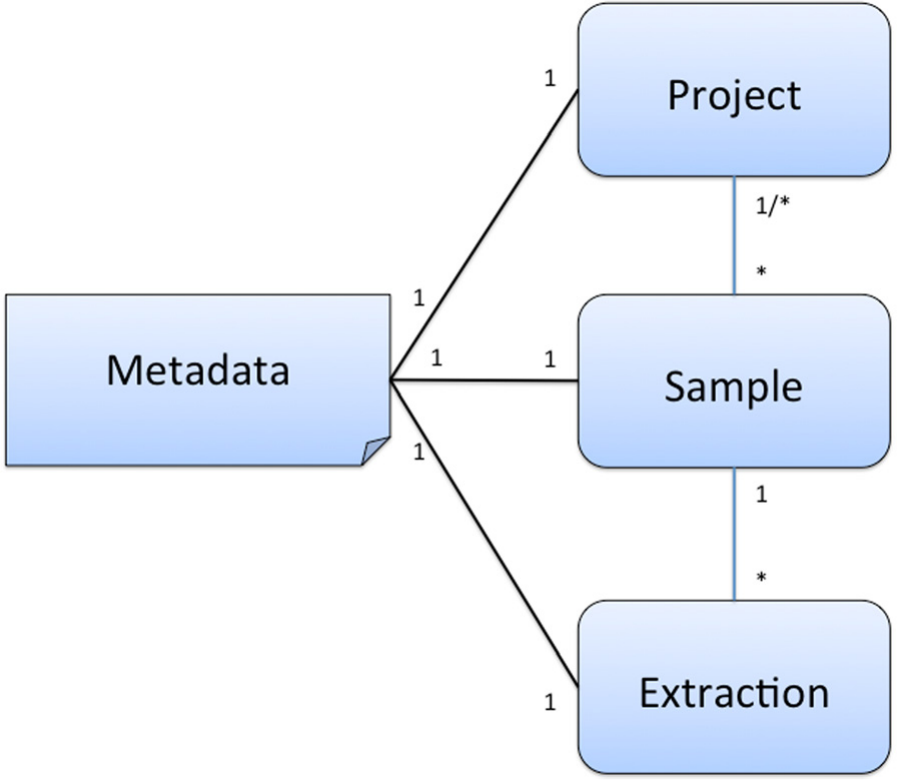
A three-tiered hierarchy allows modeling of real-life experiments, combining multiple “samples” into a “project” (or study) and allowing for different “extractions” from the sample.

Metadata is in constant flux; and while many users struggle to add it to their data, we as service providers need it to enable users to analyze their data. Think of the ability to “color” an ordination plot using user-provided data (e.g., biome, sampling location, plot name, replicate name). Rather than using a single database with a fixed schema (that would be too restrictive) or a complete schema-free approach (this would be chaotic and not offer validation), we use a hybrid approach, combining the strengths of both.This hybrid approach enables us to capture arbitrary key value pairs and have blessed and controlled subsets of the data, thus providing the best of both worlds. Figure 4 shows the general layout.

Metazen’s user interface is at [13]. Instructions for the web interfacecan be found on the Metazen site. It has a mini-FAQ, which covers such topics as creating metadata spreadsheets or preparing metadata. It can also be downloaded freely from github [15]. Installation instructions are provided there, as well. Metazen is under a GNU GPL and is platform independent.

When data is uploaded (or after the fact), a separate metadata file is created. Using a simple spreadsheet (generated by Metazen in most cases), users can capture minimal metadata required for standard compliance (currently GSC MIxS).

Users can submit their metadata spreadsheet for an entire project, with many samples and extractions, for validation. These spreadsheets can also be downloaded later to modify, update, or extend the metadata.

We use a simple schema, id | collection | key | value, to store arbitrary metadata on any data type. By (optionally) organizing metadata into collections, we can for each individual collection:

– require certain fields (keys) and

– validate the use of controlled vocabulary terms (when required for certain values) for given fields.

Metazen uses the controlled vocabularies from BioOntology for environmental package, biome, feature, and material (so values are constrained). There are two levels of validation of the metadata that is entered into Metazen. The first is where it validates that the value constrains to the data type (e.g. for numeric values we validate that inputs are integers or decimals, depending on the data type; we validate all date/time are in ISO standard, etc.). The second is where we validate that the metadata structure conforms to the template.

Metazen uses the MG-RAST API to retrieve project level metadata and our metadata template. Accessing these live resources (MG-RAST API and BioOntolgy) makes Metazen dynamically updated with changes from either of these. Metazen also uses an Oauth authentication, which provides users with private project-level metadata in addition to public information.

## Abbreviations

API: Application programming interface; GCDML: Genomic contextual data markup language; GNU: GNU’s not unix; GSC: Genomic Standards Consortium.

## Competing interests

The authors declare that they have no competing interests.

## Authors’ contributions

JMB was the main developer for Metazen; TH and AW developed the metadata collection and retrieval infrastructure in MG-RAST; TP implemented the Metazen Collect application for mobile devices; EMG wrote the manuscript and evaluated the user interface; FM conceived of the project, and he and AW provided overall and technical management, respectively. All authors read and approved the final manuscript.
